# Entropy Analysis of Carbon Nanotubes Based Nanofluid Flow Past a Vertical Cone with Thermal Radiation

**DOI:** 10.3390/e21070642

**Published:** 2019-06-28

**Authors:** Muhammad Ramzan, Mutaz Mohammad, Fares Howari, Jae Dong Chung

**Affiliations:** 1 Department of Computer Science, Bahria University, 44000 Islamabad, Pakistan; 2 Department of Mechanical Engineering, Sejong University, Seoul 143-747, Korea; 3 Department of Mathematics & Statistics, College of Natural and Health Sciences, Zayed University, 144543 Abu Dhabi, UAE; 4 College of Natural and Health Sciences, Zayed University, 144543 Abu Dhabi, UAE

**Keywords:** nanofluid, carbon nanotubes (SWCNTs and MWCNTs), solutal stratification, bioconvection, entropy generation

## Abstract

Our objective in the present study is to scrutinize the flow of aqueous based nanofluid comprising single and multi-walled carbon nanotubes (CNTs) past a vertical cone encapsulated in a permeable medium with solutal stratification. Moreover, the novelty of the problem is raised by the inclusion of the gyrotactic microorganisms effect combined with entropy generation, chemical reaction, and thermal radiation. The coupled differential equations are attained from the partial differential equations with the help of the similarity transformation technique. The set of conservation equations supported by the associated boundary conditions are solved numerically with the bvp4c MATLAB function. The influence of numerous parameters on the allied distributions is scrutinized, and the fallouts are portrayed graphically in the analysis. The physical quantities of interest including the skin friction coefficient and the rate of heat and mass transfers are evaluated versus essential parameters, and their outcomes are demonstrated in tabulated form. For both types of CNTs, it is witnessed that the velocity of the fluid is decreased for larger values of the magnetic and suction parameters. Moreover, the value of the skin friction coefficient drops versus the augmented bioconvection Rayleigh number. To corroborate the authenticity of the presented model, the obtained results (under some constraints) are compared with an already published paper, and excellent harmony is achieved in this regard.

## 1. Introduction

Nanofluid, characterized by copious attractive features, including outstanding chemical and mechanical steadiness, significant improvement in thermal conductivity, etc. [1], is found to serve in a number of engineering applications, for example fuel-cells [2], porous materials [3], petroleum engineering [4], and biotechnology [5,6], among others. The pioneering work was done by Choi and Eastman [7] who found that thermal conductivity of the base fluid will increase from the insertion of metallic particles. This was followed by a study by Buongiorno [8] who studied the features of Brownian motion and thermophoresis in nanofluids. Later, Makinde and Aziz [9] deliberated on the flow of Newtonian fluid past a convectively heated surface. The flow of 3D couple stress nanofluid past an exponentially stretching surface associated with zero mass flux at the surface and convective boundary conditions was deliberated by Ramzan et al. [10]. Farooq et al. [11] examined Newtonian fluid flow analytically over an exponentially stretching sheet under the influence of magneto hydrodynamics using the optimal homotopy analysis method. The nanofluid flows containing carbon nanotubes (CNTs) over a cone and an inclined permeable plate were studied numerically by Reddy et al. [12,13]. Sreedevi et al. [14] found a numerical solution for CNTs amalgamated nanofluid flow past a vertical cone with a convective boundary condition. The aqueous-silver non-Darcy Poiseuille nanofluid flow with entropy generation past a permeable media was studied by Shehzad et al. [15]. A few recent investigations highlighting nanofluid flows may be found in References [16,17,18].

CNTs are the hexagonal structure of carbon atoms that are rolled in a cylindrical shape. Carbon nanotubes possess unique features like corrosion resistance, high thermal conductivity, and exceptional strength [19]. Owing to these remarkable characteristics, CNTs are useful in numerous applications like nanotubes transistors, microwave amplifier, solar cells, chemical sensors, optics, drug delivery, prostheses, pharmacogenomics, and many other fields of engineering and material science [20,21,22]. Carbon nanotubes are labeled as multi-walled carbon nanotubes (MWCNTs) and single-walled carbon nanotubes (SWCNTs). Iijima [23] discovered carbon nanotubes in 1991. He first investigated MWCNTs utilizing the Krastschmer and Huffman method. This was followed by another exploration in 1993 by Bethune [24] who introduced the concept of SWCNTs. SWCNTs are comprised of carbon nanotubes with a diameter of 1 nm whereas MWCNTs is a collection of 2–50 carbon nanotubes with 0.34 nm spacing. Abundant studies may be found in the literature to highlight different aspects of CNTs. Ramasubramaniam et al. [25] found that single-walled CNTs are helpful in improving electrical conductivity. The idea of improved thermal conductivity using composite nanotubes was introduced by Xue [26]. Muhammad et al. [27] inspected the rotating flow of carbon nanotubes under the influence of heat generation/absorption and nonlinear thermal radiation past a linearly stretching surface. The flow of 3D viscous nanofluid containing CNTs with quartic chemical reaction and entropy generation analysis is expressed numerically by Kumar et al. [28]. The aqueous based nanofluid Darcy-Forchheimer 3D flow comprising CNTs past a permeable surface was examined analytically by Muhammad et al. [29]. The flow problem in Reference [29] is extended to homogeneous-heterogeneous reactions associated with convective boundary conditions is discussed by Alshomrani and Ullah [30]. Recent explorations studying CNTs nanofluid flow may be found in References [31,32,33] and those contained therein.

The aforementioned literature review reveals that abundant articles are available addressing the topic of nanofluid. But this subject gets narrower once we talk about nanofluid flow over a cone with nanotubes inserted into it. Furthermore, this exploration becomes unique when the above-mentioned characteristics are supported by entropy generation and gyrotactic microorganisms (see Table 1). The numerical solution of the problem is acquired with requisite discussion of plotted illustrations of involved parameters versus associated distributions.

## 2. Mathematical Modeling

**Figure 1:**
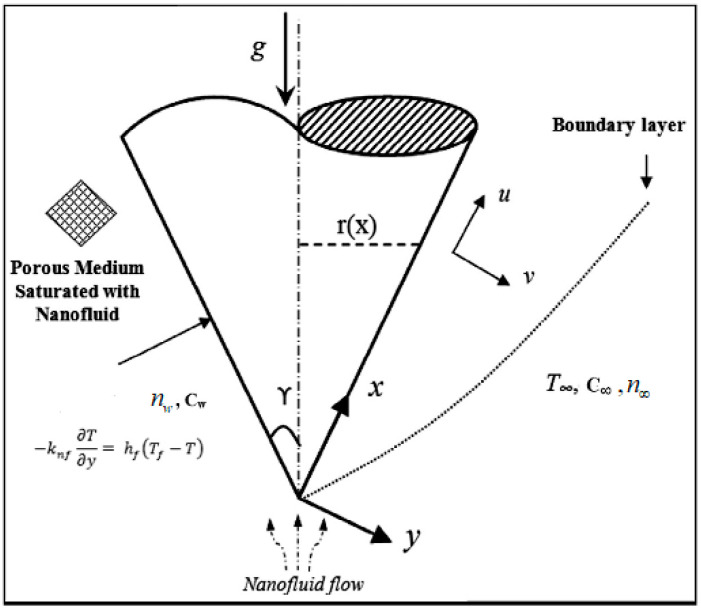
Physical model of the problem.

Let us assume a 2D aqueous fluid flow amalgamated with carbon nanotubes past a vertical cone in an absorbent media. The analysis is accompanied by solutal stratification, chemical reaction, and entropy generation (see Figure 1).

The flow of the fluid is along the *x*-axis past the cone surface. Along the *y*-axis, a magnetic field with strength *B_0_* is enforced. The fluid is an aqueous based nanofluid containing both types of CNTs, whose thermo-physical characteristics are defined in Table 2. The governing system of equations representing the presented model are as follows [14]:

(1) $$\frac{\partial (ru)}{\partial x}+\frac{\partial (rv)}{\partial y}=0,$$

(2) $$\begin{array}{c} u\frac{\partial u}{\partial x}+v\frac{\partial u}{\partial y}=\frac{\mu_{nf}}{\rho_{nf}}\frac{\partial^{2}u}{\partial y^{2}}-\frac{\mu_{nf}}{\rho_{nf}}\frac{1}{K}u+g\left[\beta (T-T_{\infty })-\beta^{*}(C-C_{\infty })-\beta^{*}\gamma (n-n_{\infty })\Delta \rho \right]\cos\gamma \\ -\frac{\sigma B_{0}{}^{2}}{\rho_{nf}}u, \end{array}$$

(3) $$u\frac{\partial T}{\partial x}+v\frac{\partial T}{\partial y}=\alpha_{nf}\frac{\partial^{2}T}{\partial y^{2}}-\frac{1}{{\left(\rho c_{p}\right)}_{nf}}\frac{\partial q_{r}}{\partial y},$$

(4) $$u\frac{\partial C}{\partial x}+v\frac{\partial C}{\partial y}=D_{m}\frac{\partial^{2}C}{\partial y^{2}}-K_{r}(C-C_{\infty }),$$

(5) $$u\frac{\partial n}{\partial x}+v\frac{\partial n}{\partial y}+\frac{bW_{c}}{C_{w}-C_{0}}\frac{\partial }{\partial y}\left(n\frac{\partial C}{\partial y}\right)=D_{n}\frac{\partial^{2}n}{\partial y^{2}},$$

with the corresponding boundary conditions

(6) $$\begin{array}{l} v=V_{1},\;u=0,-k_{nf}\frac{\partial T}{\partial y}=h_{f}(T_{f}-T),C=C_{w}=C_{0}+dx,\;n=n_{w},\;\mathrm{at}\;y=0,\\ u\to 0,\;T\to T_{\infty },\;C\to C_{\infty }=C_{0}+ex,\;n\to n_{\infty },\;\mathrm{as}\;y\to \infty . \end{array}$$

The hypothetical relations are characterized as follows:

(7) $$\mu_{nf}=\frac{\mu_{f}}{{\left(1-\phi \right)}^{2.5}},\;v_{nf}=\frac{\mu_{nf}}{\rho_{nf}},$$

(8) $$\rho_{nf}=(1-\phi )\rho_{f}+\phi \rho_{CNT},\;\alpha_{nf}=\frac{k_{nf}}{\rho_{nf}{\left(c_{p}\right)}_{nf}},$$

(9) $$\frac{k_{nf}}{k_{f}}=\frac{\left(1-\phi )+2\phi \frac{k_{CNT}}{k_{CNT}-k_{f}}\ln(\frac{k_{CNT}+k_{f}}{2k_{f}}\right)}{\left(1-\phi )+2\phi \frac{k_{f}}{k_{CNT}-k_{f}}\ln(\frac{k_{CNT}+k_{f}}{2k_{f}}\right)}.$$

Using the similarity transformations

(10) $$\begin{array}{cc} \eta = & \frac{y}{x}Ra_{x}{}^{1/4},\;\Psi =\alpha Ra_{x}{}^{1/4}f(\eta ),\;\theta (\eta )=\frac{T-T_{\infty }}{T_{w}-T_{\infty }},\;\\ & g(\eta )=\frac{C-C_{\infty }}{C_{w}-C_{0}},\;h(\eta )=\frac{n-n_{\infty }}{n_{w}-n_{\infty }}, \end{array}$$

Equation (1) is impartially fulfilled and Equations (2) to (6) obtain

(11) $$\begin{array}{cc} f^{\prime \prime \prime }+\frac{1}{\mathrm{Pr}}{\left(1-\phi \right)}^{2.50}(1-\phi +\phi & \frac{\rho_{CNT}}{\rho_{f}})\left\{3ff^{\prime \prime }-\frac{1}{2}f^{\prime^{2}}\right\}-k_{1}f^{\prime }-{\left(1-\phi \right)}^{2.5}Mf{}^{\prime }\\ & +{\left(1-\phi \right)}^{2.50}(1-\phi +\phi \frac{\rho_{CNT}}{\rho_{f}})[\theta -N_{r}g-R_{b}h]=0, \end{array}$$

(12) $$\frac{k_{nf}}{k_{f}}\left(1+R_{d}\right)\theta {}^{\prime \prime }+\frac{3}{4}\left[1-\phi +\phi \frac{{\left(\rho C_{p}\right)}_{CNT}}{{\left(\rho C_{p}\right)}_{f}}\right]f\theta {}^{\prime }=0,$$

(13) $$g{}^{\prime \prime }+\frac{3}{4}S_{c}fg{}^{\prime }-S_{c}nf{}^{\prime }-C_{r}g=0,$$

(14) $$h{}^{\prime \prime }+\frac{3}{4}L_{b}fh{}^{\prime }-P_{e}(h{}^{\prime }g{}^{\prime }+(h+\delta )g{}^{\prime \prime })=0,$$

and the boundary conditions (6) take the form

(15) $$\begin{array}{l} f(0)=V_{0},\;f^{\prime }(0)=0,\;\frac{k_{nf}}{k_{f}}\theta {}^{\prime }(0)=-B_{1}(1-\theta (0)),\;g(0)=1-n,\;h(0)=1,\\ f{}^{\prime }(\infty )\to 0,\;\theta (\infty )\to 0,\;g(\infty )\to 0,\;h(\infty )\to 0. \end{array}$$

In the aforementioned equations, the dimensionless parameters are given by:

(16) $$\begin{array}{l} \mathrm{Pr}=\frac{\upsilon_{f}}{\alpha },\;k_{1}=\frac{x^{2}}{KRa_{x}{}^{1/2}},\;M=\frac{\sigma B_{0}{}^{2}x^{2}}{\mu_{f}Ra_{x}{}^{1/2}},\;S_{c}=\frac{\alpha }{D_{m}},\;n=\frac{e}{d},\\ L_{b}=\frac{\alpha }{D_{n}},\;R_{d}=\frac{16T_{\infty }{}^{3}\sigma^{*}}{3k^{*}k_{nf}},\;N_{r}=\frac{\beta^{*}(C_{w}-C_{0})}{\beta (T_{f}-T_{\infty })},R_{b}=\frac{\beta^{*}\gamma \Delta \rho \Delta n_{w}}{\beta (T_{f}-T_{\infty })},\;\\ C_{r}=\frac{K_{r}x^{2}}{D_{m}Ra_{x}{}^{1/2}},B_{1}=\frac{h_{f}x}{Ra_{x}{}^{1/4}k_{f}},\;P_{e}=\frac{bW_{c}}{D_{n}},\;\delta =\frac{n_{\infty }}{n_{w}-n_{\infty }}. \end{array}$$

The physically essential quantities, i.e., the skin friction, rate of heat and mass transfers, and local density of motile microorganisms, are appended below:

(17) $$C_{f}=\frac{\tau_{w}}{\rho U_{\infty }{}^{2}},Nu_{x}=\frac{xq_{w}}{k_{f}(T_{w}-T_{\infty })},\;Sh_{x}=\frac{xq_{m}}{D_{m}(C_{w}-C_{0})},Nn_{x}=\frac{xq_{n}}{D_{n}(n_{w}-n_{\infty })}.$$

The aforementioned physical quantities in dimensionless form are appraised as follows:

(18) $$\begin{array}{l} C_{f}Ra_{x}{}^{1/4}=\frac{1}{{\left(1-\phi \right)}^{2.5}}f\prime \prime (0),\\ Nu_{x}Ra_{x}{}^{-1/4}=-\frac{k_{nf}}{k_{f}}(1+R_{d})\theta^{\prime }(0),\\ Sh_{x}Ra_{x}{}^{-1/4}=-g{}^{\prime }(0),\\ Nn_{x}Ra_{x}{}^{-1/4}=-h{}^{\prime }(0). \end{array}$$

Table 3 depicts a comparison with Khan et al. [34] for varied estimates of ϕ in limiting case. An outstanding matching in both results is achieved. This reflects the corroboration of the presented outcomes.

### Entropy Generation

The entropy generation of the presented model is specified as follows:

(19) $$\begin{array}{c} S_{gen}{}^{{}^{\prime \prime \prime }}=\underset{HFI}{\underbrace{\frac{k_{nf}}{T_{\infty }{}^{2}}\left[1+\frac{16T_{\infty }{}^{3}\sigma^{*}}{3k^{*}k_{nf}}\right]{\left(\frac{\partial T}{\partial y}\right)}^{2}}}+\underset{FFI}{\underbrace{\frac{\mu_{nf}}{T_{\infty }}{\left(\frac{\partial u}{\partial y}\right)}^{2}+\frac{\sigma }{T_{\infty }}B_{0}{}^{2}u^{2}+\frac{\mu_{nf}}{T_{\infty }K}u^{2}}}\\ +\underset{Diffusive\;irreversibility}{\underbrace{\frac{RD}{C_{\infty }}{\left(\frac{\partial C}{\partial y}\right)}^{2}+\frac{RD}{T_{\infty }}\left(\frac{\partial T}{\partial y}\right)\left(\frac{\partial C}{\partial y}\right)}}. \end{array}$$

In Equation (19), entropy is comprised of three terms, namely (*i*) HFI (heat transfer irreversibility), (*ii*) FFI (fluid friction irreversibility), and (*iii*) diffusion irreversibility. The entropy generation $N_{G}$ is defined as:

(20) $$N_{G}=\frac{S^{{}^{\prime \prime \prime }}{}_{gen}}{S_{0}{}^{{}^{\prime \prime \prime }}},$$

where $S^{{}^{\prime \prime \prime }}{}_{gen}$ and $S_{0}{}^{{}^{\prime \prime \prime }}$ characterize the entropy generation rate and characteristic entropy generation rate, respectively, such that

(21) $$\begin{array}{c} N_{G}=\frac{k_{nf}}{k_{f}}\left(1+R\right)Ra_{x}\theta {{}^{\prime }}^{2}+\frac{1}{{\left(1-\phi \right)}^{2.5}}\frac{BrRa_{x}}{\alpha }(f{{}^{\prime \prime }}^{2}+k_{1}f{{}^{\prime }}^{2})+\frac{Ra_{x}BrM}{\alpha }f{{}^{\prime }}^{2}\\ +\lambda {\left(\frac{\zeta }{\alpha }\right)}^{2}Ra_{x}g{{}^{\prime }}^{2}+\frac{\zeta }{\alpha }Ra_{x}\lambda \theta {}^{\prime }g{}^{\prime }. \end{array}$$

Parameter used in above equation are define as,

(22) $$\alpha =\frac{\Delta T}{T_{\infty }},Br=\frac{\mu_{f}u_{w}}{k_{f}\Delta T},\zeta =\frac{\Delta C}{C_{\infty }},\lambda =\frac{RDC_{\infty }}{k_{f}}.$$

## 3. Results and Discussion

**Figure 2:**
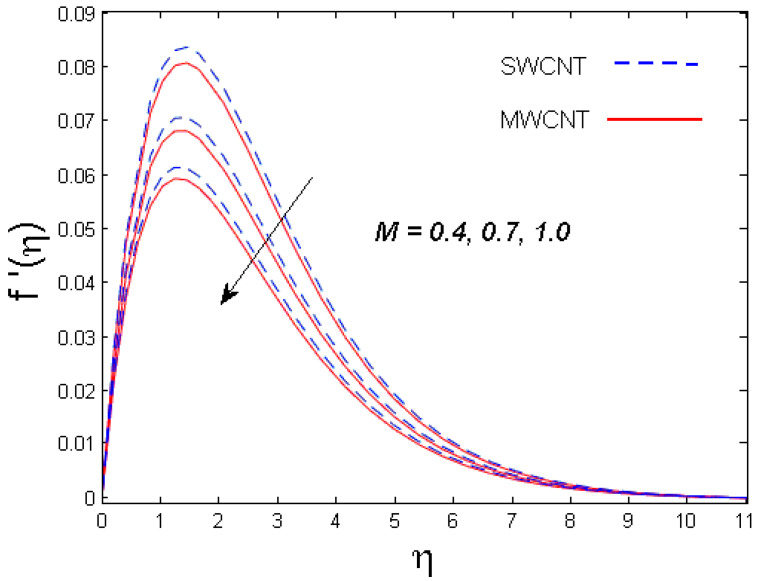
Consequence of M on $f{}^{\prime }(\eta )$.

**Figure 3:**
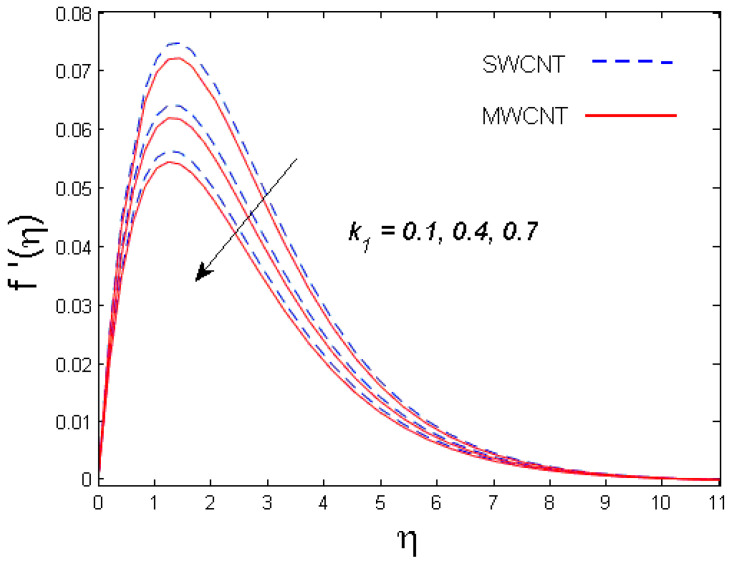
Consequence of $k_{1}$ on $f{}^{\prime }(\eta )$.

**Figure 4:**
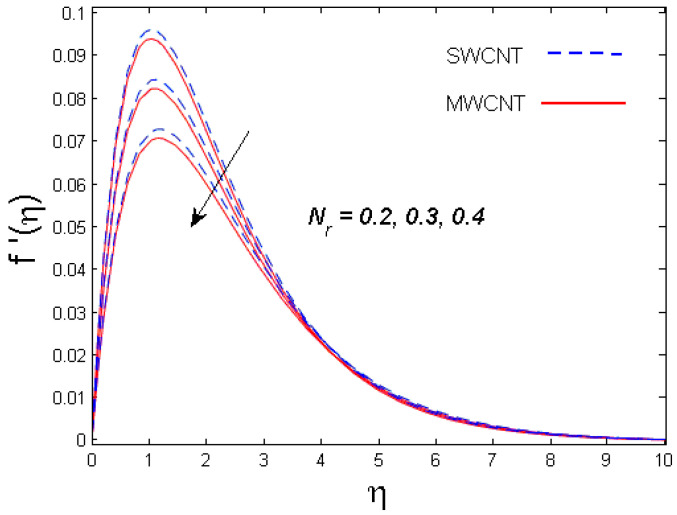
Consequence of $N_{r}$ on $f{}^{\prime }(\eta )$.

**Figure 5:**
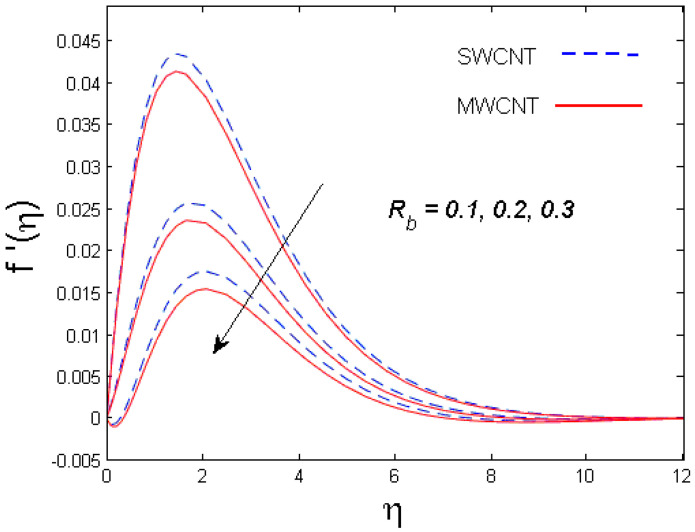
Consequence of $R_{b}$ on $f{}^{\prime }(\eta )$.

**Figure 6:**
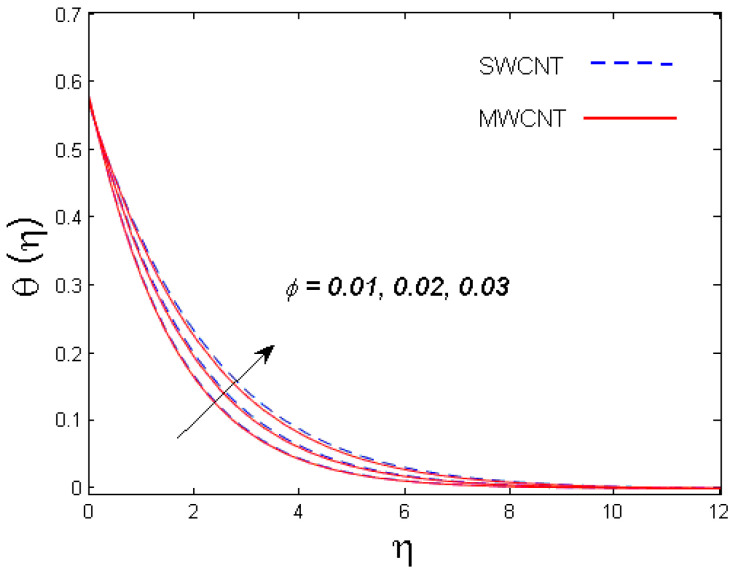
Consequence of ϕ on θ(η).

**Figure 7:**
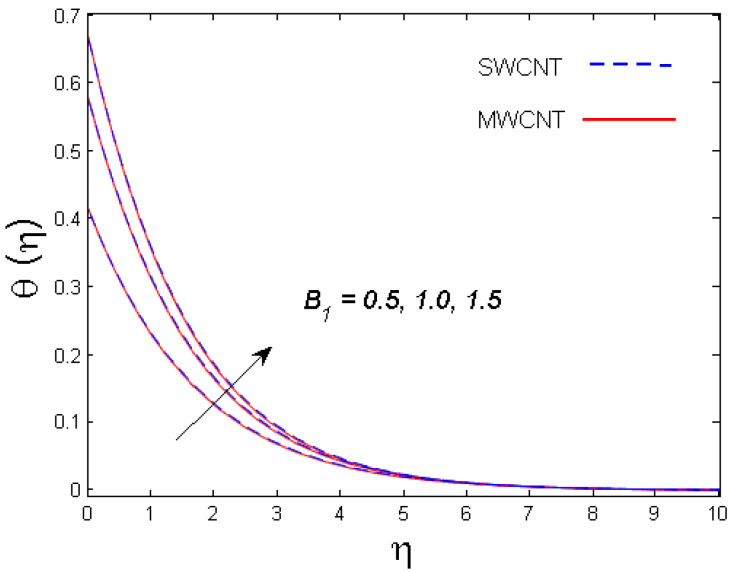
Consequence of $B_{1}$ on θ(η).

**Figure 8:**
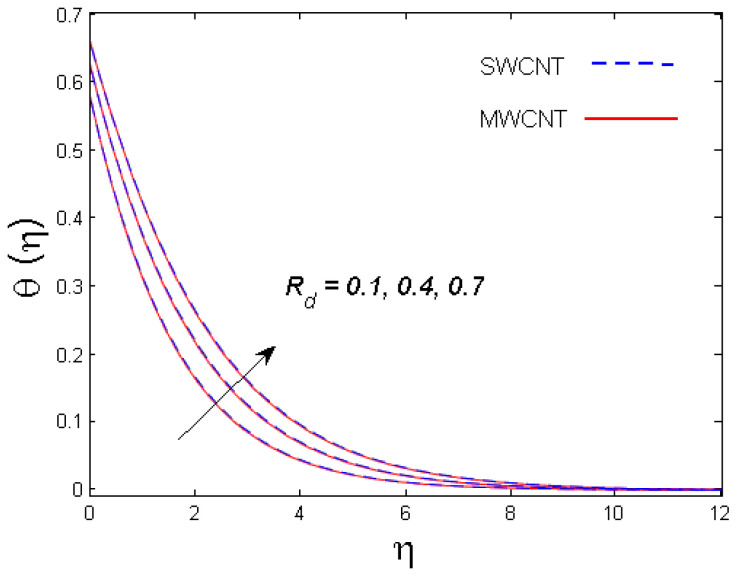
Consequence of $R_{d}$ on θ(η).

**Figure 9:**
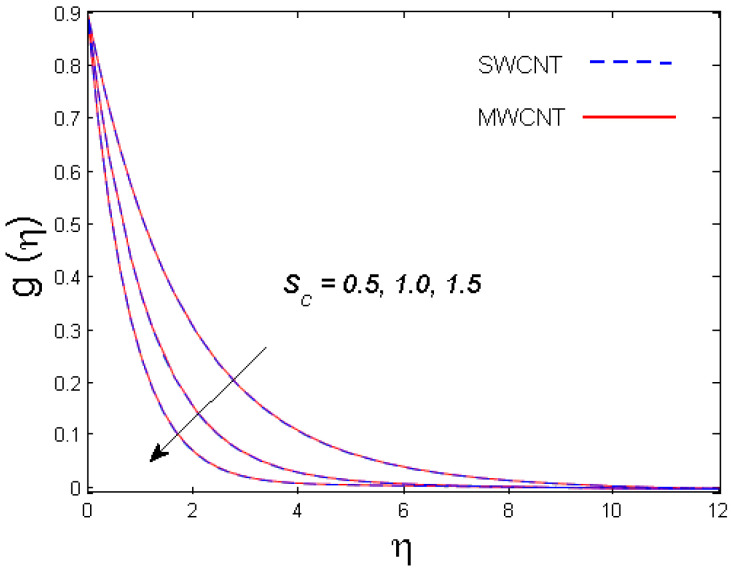
Consequence of $S_{c}$ on g(η).

**Figure 10:**
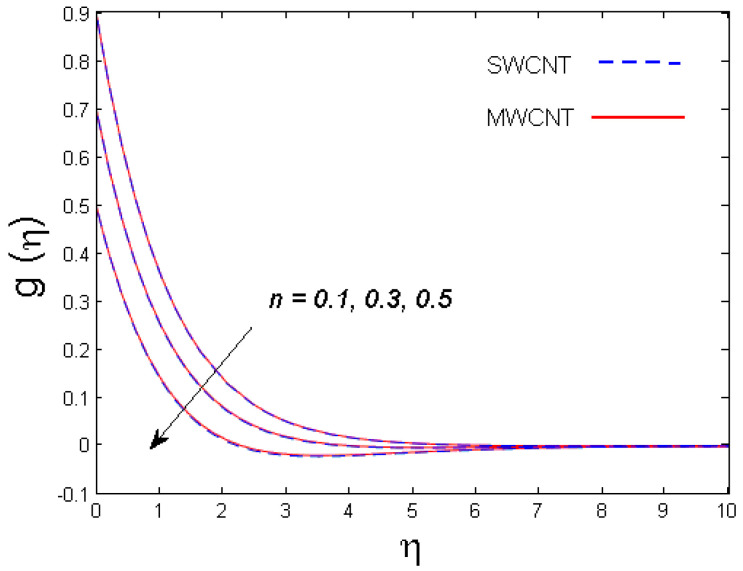
Consequence of n on g(η).

**Figure 11:**
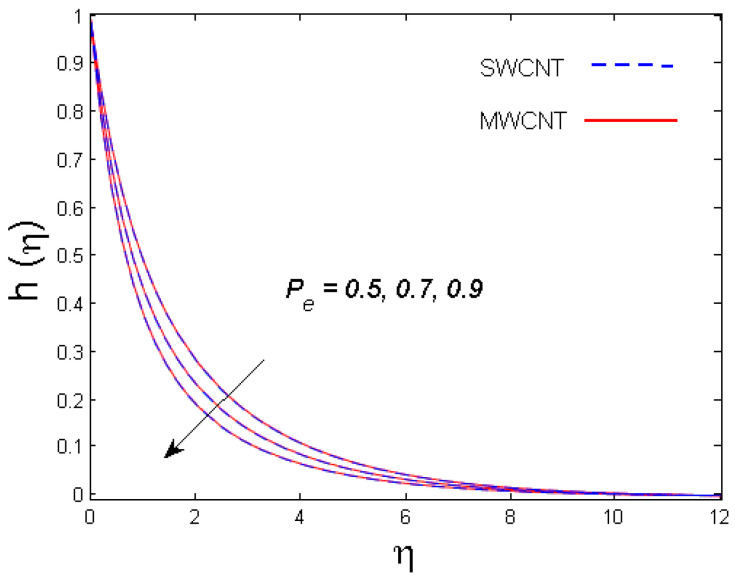
Consequence of $P_{e}$ on h(η).

**Figure 12:**
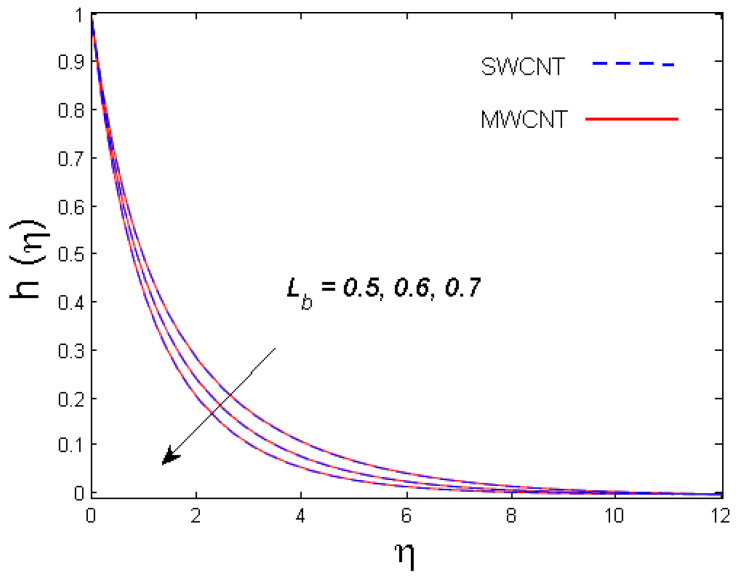
Consequence of $L_{b}$ on h(η).

**Figure 13:**
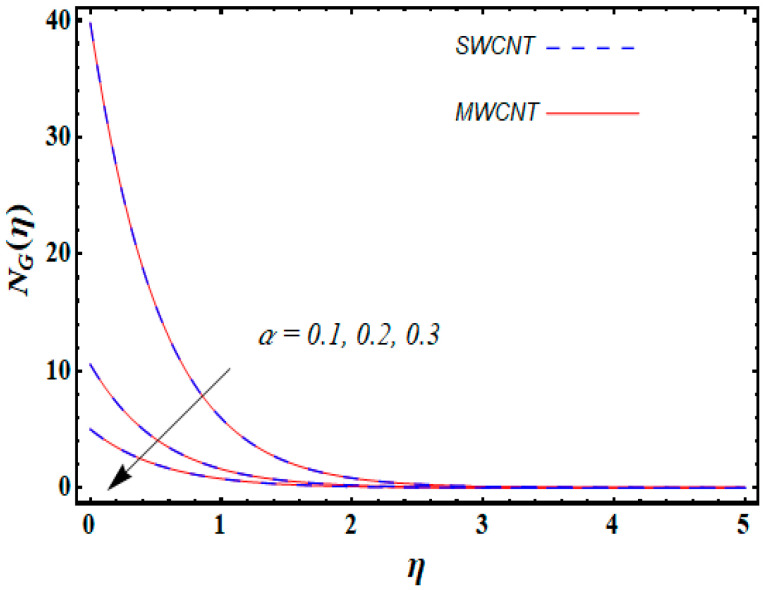
Consequence of α on $N_{G}(\eta )$.

**Figure 14:**
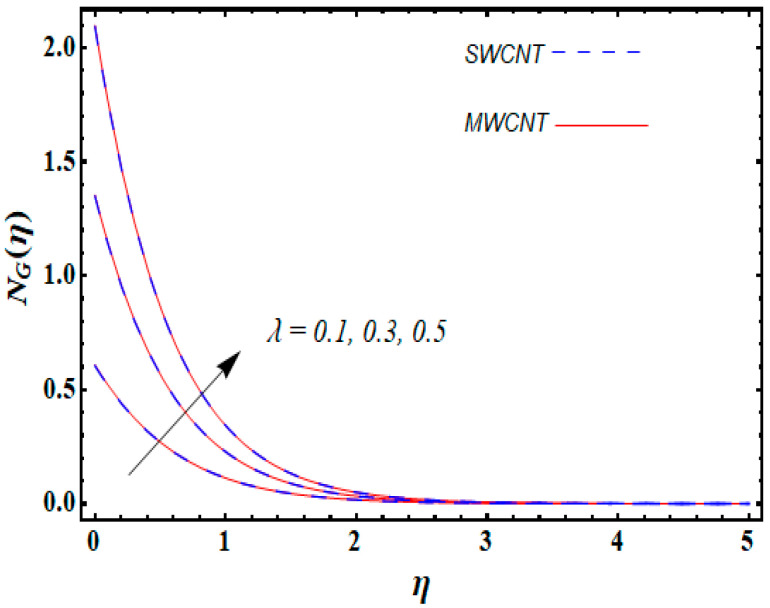
Consequence of λ on $N_{G}(\eta )$.

This section is devoted to comprehend the discussion of the graphical illustrations. The impressions of the miscellaneous parameters on entangled profiles are given in Figure 2, Figure 3, Figure 4, Figure 5, Figure 6, Figure 7, Figure 8, Figure 9, Figure 10, Figure 11, Figure 12, Figure 13 and Figure 14. The numerical values of the parameters used are taken to be fixed as: $\phi =0.01,\;N_{r}=P_{e}=k_{1}=0.5=L_{b},\;S_{c}=B_{1}=1.0=M=V_{0},\;R_{b}=n=R_{d}=0.1=C_{r}=\delta \;\mathrm{and}\;\mathrm{Pr}=6.2$ unless otherwise stated. The ranges of parameters defined in the figures are 0.4≤M≤1.0, $0.1\leq k_{1}\leq 0.7,$ $0.2\leq N_{r}\leq 0.4,$ $0.1\leq R_{b}\leq 0.3,$ 0.01≤ϕ≤0.03, $0.5\leq B_{1}\leq 1.5,$ $0.1\leq R_{d}\leq 0.7,$ $0.5\leq S_{c}\leq 1.5,$ 0.1≤n≤0.5, $0.5\leq P_{e}\leq 0.9,$ $0.5\leq L_{b}\leq 0.7,$ 0.1≤α≤0.3, and 0.1≤λ≤0.5.

### 3.1. Velocity Profile

The trend of axial velocity versus different parameters’ effects is described in Figure 2, Figure 3, Figure 4 and Figure 5. Figure 2 depicts the impact of the magnetic parameter M on the velocity field. The velocity of the fluid diminishes for increasing values of M. This is because of the strong Lorentz force that presents resistance to the fluid’s movement that eventually lowers the fluid’s movement. In Figure 3, the consequence of porous parameter $k_{1}$ on the velocity profile is sketched. It is understood that the velocity is a decreasing function of $k_{1}$. Physically, more resistance against the fluid’s movement is witnessed due to the augmented thickness of the permeable medium that results in feeble fluid velocity. The impact of the buoyancy ratio parameter $N_{r}$ and the bioconvection Rayleigh number $R_{b}$ on the velocity profile for both CNTs is depicted in Figure 4 and Figure 5, respectively. It is witnessed that the velocity profile declines with increasing values of $N_{r}$ and $R_{b}$. Higher values of the buoyancy ratio parameter mean an increase in the number of CNTs immersed into the aqueous solution, which increases the viscosity of the fluid and results in a decrease in fluid’s velocity. Similarly, the velocity of the fluid is affected by the growth in the bioconvection Rayleigh number. This is due to the inertia force of the fluid motion being surpassed by the bioconvection. There is a decrease in speed of roughly 15.48% with an approximate increase in $R_{b}$ of 400% [35].

### 3.2. Temperature Profile

The outcome of solid volume fraction ϕ on the temperature field is evident in Figure 6. The temperature profile is enhanced with increasing estimates of the solid volume fraction of the nanoparticles. It is also understood that the thermal boundary layer thickness is enhanced by augmenting the estimation of the solid volume fraction ϕ for both nanotubes. This is all because of the enhancement in thermal conductivity of CNTs with solid volume fraction that becomes the main cause for augmented temperature. The effect of Biot number $B_{1}$ is studied in Figure 7. It is perceived that with an upsurge in $B_{1}$, temperature distribution escalates for both SWCNT and MWCNT. Physically, larger estimates of $B_{1}$ means more thermal resistance inside the cone in comparison to the boundary layer; consequently, a higher temperature of the fluid in the boundary layer area is witnessed. Figure 8 shows the influence of radiation parameter $R_{d}$ on temperature profile. It was determined that larger values of $R_{d}$ result in more energy being produced, which eventually raises the temperature of the fluid.

### 3.3. Concentration Profile

The impact of numerous parameters on the concentration field is presented in Figure 9 and Figure 10. Figure 9 depicts the influence of Schmidt number $S_{c}$ on concentration distribution for both nanotubes. It is understood from the figure that the concentration field is a diminishing function of $S_{c}$. Since the Schmidt number is the proportion of kinematic viscosity and the molecular diffusion coefficient, higher values of $S_{c}$ leads to a reduced molecular diffusion that ultimately lowers the concentration of the fluid. In Figure 10, the graph of concentration profile versus the solutal stratification n is depicted. It is clear that for improved values of n, the concentration of the fluid is diminished for both SWCNT and MWCNT nanotubes. In actuality, the lowering of concentration field is due of the concentration differences between the ambient fluid and the surface of the cone.

### 3.4. Density of Motile Microorganism Profile

Figure 11 and Figure 12 demonstrate the impacts of the bioconvection Péclet number and the bioconvection Lewis number on the density of motile microorganisms, respectively. It is observed that motile microorganisms decrease for both bioconvection Péclet number and bioconvection Lewis numbers. Indeed, higher estimates of the bioconvection Péclet and bioconvection Lewis numbers result in a decline in the microorganism diffusion, which ultimately results in the decay of the density and boundary layer thickness of motile microorganisms.

### 3.5. Entropy Generation

From Figure 13, it is seen that increasing the temperature difference parameter α decreases the entropy generation number $N_{G}$ for both nanoparticles. Similarly, the local entropy generation increases for growing estimates of the diffusive constant parameter λ for both SWCNT and MWCNT, which is displayed in Figure 14.

Table 4 shows that the skin friction coefficient is enhanced with increase in the values of the solid volume fraction of nanoparticles and suction parameter, while it declines for higher values of the porous medium, magnetic parameter, and bioconvection Rayleigh number. Table 5 demonstrates the numerical values of the Nusselt number for different varying parameters. It is found that the Nusselt number rises for larger estimates of solid volume fraction, radiation parameter, and Biot number, and decreases when the values of the magnetic parameter are increased. Table 6 displays the numerical value of the Sherwood number for varied parameters. The Sherwood number boosted with an increase in the values of the chemical reaction parameter and the Schmidt number, while it decreases with an increase in the values of the concentration stratification and buoyancy ratio parameter. Table 7 depicts the numerical value of motile density number versus different parameters. The motile density number increases for larger estimates of the Péclet number and microorganism concentration difference parameter and decreases for increasing values of the Rayleigh number.

## 4. Final Remarks

The flow of water based carbon nanotubes (SWCNT and MWCNT) fluid past a cone erected vertically is discussed numerically. The analysis is performed in the presence of motile organisms with solutal stratification in spongy media. Furthermore, the attributes of thermal radiation and chemical species are explored in the presence of entropy generation. The main outcomes of the analysis are:

➢ The velocity of the fluid diminishes with increasing values of the magnetic and suction parameters in the case of both nanotubes.
➢ The fluid’s concentration is diminished for both SWCNT and MWCNT nanotubes versus higher values of solutal stratification.
➢ When increasing the temperature difference parameter, the entropy generation number decreases for both nanoparticles.
➢ The Sherwood number increases with increasing values of the chemical reaction parameter and the Schmidt number, while it decreases with increasing estimates of solutal stratification.
➢ The motile density number decreases with increasing values of the Péclet number.
➢ The skin friction coefficient increases for the suction parameter while decreasing for the bioconvection Rayleigh number.
➢ It is found that the Nusselt number increases with an increase in the estimates of solid volume fraction, radiation parameter, and Biot number, whereas it decreases with increasing values of the magnetic parameter.

## Figures and Tables

**Table 1:** The studies on nanoliquid flow comprising carbon nanotubes (CNTs).

Authors	CNTs SWCNTs/MWCNTs	Entropy Generation	Gyrotactic Microorganisms	Flow over a Cone
Reddy et al. [12]	√	×	×	√
Reddy et al. [13]	√	×	×	×
Sreedevi et al. [14]	√	×	×	√
Kumar et al. [28]	√	√	×	×
Muhammad et al. [29]	√	×	×	×
Alshomrani & Ullah [30]	√	×	×	×
Lu et al. [31]	√	×	×	×
Ramzan et al. [32]	√	√	×	×
Lu et al. [33]	√	√	×	×
Present	√	√	√	√

(√) means effect is present, and (×) means effect is absent.

**Table 2:** Values of physical features of nanoparticles and water [14].

Physical Attributes	Liquid	Nanoparticles	
	H_2_O	SWCNTs	MWCNTs
C_p_(J/kg K)	4179	425	796
ρ (kg/m^3^)	997	2600	1600
*k* (W/mK)	0.613	6600	3000

Multi-walled carbon nanotubes (MWCNTs) and single-walled carbon nanotubes (SWCNTs).

**Table 3:** Evaluation of the presented model with Khan et al. [34] in limiting case.

ϕ	$f^{\prime \prime }(0)$				$-\theta^{\prime }(0)$			
	Khan et al. [34]		Existing Results		Khan et al. [34]		Existing Results	
	SWCNT	MWCNT	SWCNT	MWCNT	SWCNT	MWCNT	SWCNT	MWCNT
0.01	0.33894	0.33727	0.338910	0.337270	1.10553	1.07905	1.105710	1.079040
0.1	0.40811	0.39008	0.408120	0.390070	4.80627	4.27718	4.806290	4.277160
0.2	0.50452	0.46466	0.504530	0.464660	12.30317	10.56783	12.30352	10.56796

**Table 4:** Numerical value of $\frac{1}{{\left(1-\phi \right)}^{2.5}}f{}^{\prime \prime }(0)$.

ϕ	$k_{1}$	$V_{0}$	$R_{b}$	M	$\frac{1}{{\left(1-\phi \right)}^{2.5}}f^{\prime \prime }(0)$	
					SWCNTs	MWCNTs
0.1	0.5	1.0	0.1	1.0	1.11420	0.57810
0.2					1.23160	1.00280
0.3					1.47580	1.11930
	0.2				1.29720	1.26970
	0.3				1.22650	1.16470
	0.4				1.16610	1.07700
		0.5			1.04840	0.87042
		0.6			1.07060	0.89693
		0.7			1.09350	0.92345
			0.2		1.10830	0.94259
			0.3		1.05020	0.88198
			0.4		0.99171	0.82097
				0.5	1.46240	1.25400
				0.6	1.38720	1.19320
				0.7	1.32120	1.13840

**Table 5:** Numerical value of $-\frac{k_{nf}}{k_{f}}(1+R_{d})\theta^{\prime }(0)$.

ϕ	$R_{d}$	$B_{1}$	M	$-\frac{k_{nf}}{k_{f}}(1+R_{d})\theta^{\prime }(0)$	
				SWCNTs	MWCNTs
0.01	0.1	1.0	1.0	0.45621	0.45560
0.02				0.46268	0.46097
0.03				0.47205	0.46855
	0.2			0.47736	0.47659
	0.3			0.49760	0.49666
	0.4			0.51704	0.51594
		0.5		0.31751	0.31731
		0.7		0.38387	0.38351
		1.0		0.45621	0.45560
			1.0	0.45621	0.45560
			2.0	0.45279	0.45238
			3.0	0.45094	0.45063

**Table 6:** Numerical values of $-g^{\prime }(0)$.

$S_{c}$	$C_{r}$	n	$N_{r}$	$-g^{\prime }(0)$	
				SWCNTs	MWCNTs
0.1	0.1	0.1	0.5	0.31891	0.31882
0.5				0.50221	0.50155
0.9				0.74207	0.74087
	0.1			0.80642	0.80511
	0.2			0.88714	0.88613
	0.3			0.95695	0.95612
		0.2		0.73573	0.73379
		0.3		0.66795	0.66532
		0.4		0.60326	0.59988
			0.6	0.79903	0.79771
			0.7	0.79130	0.78997
			0.8	0.78319	0.78185

**Table 7:** Numerical values of $Nn_{x}Ra_{x}{}^{-1/4}$.

$L_{b}$	$P_{e}$	$R_{b}$	δ	$-h^{\prime }(0)$	
				SWCNTs	MWCNTs
0.5	0.5	0.1	0.1	0.83681	0.83535
0.6				0.91358	0.91207
0.7				0.99024	0.98870
	0.1			0.48494	0.48380
	0.2			0.57267	0.57146
	0.3			0.66056	0.65927
		0.2		0.82280	0.82132
		0.3		0.80759	0.80609
		0.4		0.79092	0.78938
			0.2	0.87679	0.87530
			0.3	0.91679	0.91528
			0.4	0.95681	0.95528

## Nomenclature

***u: v***	velocity components
***x, y***	coordinate
$K_{r}$	rate of chemical reaction
K	permeability parameter
$h_{f}$	convective parameter
$B_{1}$	Boit number
$N_{r}$	buoyancy ratio parameter
$V_{0}$	suction/injection parameter
*T*, $T_{f}$	temperature
$D_{n}$	diffusivity of microorganisms
Pr	Prandtl number
$C_{p}$	specific heat
$u_{w}$	stretching velocity along *x*-direction
$B_{0}$	magnetic field of strength
$D_{m}$	Brownian diffusion coefficient
*C_f_*	surface drag force
*Nu_x_*	Nusselt number
$P_{e}$	bioconvection Péclet number
$C_{r}$	chemical reaction parameter
$S_{c}$	Schmidt number
$W_{c}$	maximum cell swimming speed
$k_{1}$	porous parameter
$R_{b}$	bioconvection Rayleigh number
$R_{d}$	radiation parameter
$Ra_{x}$	local Rayleigh number
$N_{G}$	entropy generation number
$L_{b}$	bioconvection Lewis number
M	magnetic parameter
**Greek Symbols**	
$\rho_{CNT}$, $\rho_{f}$	density
$\sigma^{*}$	Stephan-Boltzmann constant
$\mu_{nf}$, $\mu_{f}$	dynamic viscosity
$\tau_{xy}$	shear stress
$\alpha_{nf}$	modified thermal diffusivity
${\left(\rho C_{p}\right)}_{nf}$,${\left(\rho C_{p}\right)}_{f}$	heat capacity
$k_{f}$, $k_{nf}$, k	thermal conductivity
ϕ	solid volume fraction of nanofluid
η	a scaled boundary-layer coordinate
Ψ	stream function
$q_{w}(x)$	the surface heat flux of nanoliquid film
β	thermal expansion coefficient
*f*	dimensionless stream function
θ	dimensionless temperature
δ	bioconvection constant
α	temperature difference parameter
λ	diffusive constant parameter
ζ	concentration difference
